# Empathy-Mediated Narrative Reconstruction of Autobiographical Memory: An Integrative Review of Theory, Evidence, and Applications

**DOI:** 10.3390/brainsci16040429

**Published:** 2026-04-20

**Authors:** Shigetada Hiraoka, Shuzo Kumagai, Takao Yamasaki

**Affiliations:** 1 Department of Rehabilitation, Minkodo Minohara Hospital, Fukuoka 811-2402, Japan; hrca86mmo@outlook.jp; 2 Department of Research and Development, Kumagai Institute of Health Policy, Fukuoka 816-0812, Japan; kumagai.shuzo.296@m.kyushu-u.ac.jp; 3 Department of Neurology, Minkodo Minohara Hospital, Fukuoka 811-2402, Japan

**Keywords:** autobiographical memory, narrative reconstruction, empathy, memory reconsolidation, self-narrative, social cognition, aging, narrative intervention, creative storytelling

## Abstract

**Highlights:**

**What are the main findings?**

- Autobiographical memory is conceptualized as reconstructed through narrative and empathy.
- This review integrates psychological, neuroscientific, and narrative perspectives.

**What are the implications of the main findings?**

- An empathy-mediated model of identity reconstruction is proposed.
- Narrative approaches support adaptive psychological functioning.

**Abstract:**

**Background**: Autobiographical memory undergoes qualitative changes across the lifespan, influencing self-understanding, emotional regulation, and psychological adaptation. Research shows memory is a dynamic process, reconstructed through retrieval, narration, and social interaction. How narrative construction and empathic engagement shape memory reconsolidation and self-continuity remains insufficiently integrated. **Objectives**: This narrative review synthesizes theoretical, empirical, and applied findings on autobiographical memory, narrative processes, and empathy, proposing an integrative model linking memory reconsolidation, identity reconstruction, and adaptive functioning. **Methods**: A theory-oriented narrative review was conducted across psychology, neuroscience, gerontology, and narrative research, drawing on literature from PubMed, PsycINFO, Web of Science, Scopus, J-STAGE, and CiNii. Peer-reviewed empirical studies, systematic reviews, and theoretical papers were organized around three interrelated conceptual domains: (1) autobiographical memory and self-related processes, (2) neurobiological and emotional mechanisms relevant to memory updating and reconsolidation, and (3) narrative construction within empathically mediated social interaction contexts, with additional consideration of evidence from narrative-based and creative interventions. **Results**: The reviewed literature suggests that autobiographical memory functions as a plastic, socially embedded system supporting self-continuity, although the strength and consistency of evidence vary across studies and contexts. Narrativization within empathically responsive and psychologically safe contexts enhances narrative coherence, emotional integration, and perspective-taking, promoting psychological stability, although these effects are not uniformly observed across all populations and study designs. Creative narrative activities further facilitate retrieval and meaning reconstruction, extending memory updating beyond recall, while the underlying mechanisms and causal pathways remain to be fully established. **Conclusions**: We propose an empathy-mediated narrative reconstruction model in which creative activity, narration, empathic response, and retelling interact cyclically to support memory reconsolidation and self-narrative updating. By integrating cognitive, social, and creative dimensions, this model provides a theoretically grounded framework with implications for clinical, educational, gerontological, and creative applications.

## 1. Introduction

With advancing age, the ways in which autobiographical memories and everyday events are recalled change, influencing self-understanding, emotional regulation, and psychological adaptation. Understanding these changes is crucial not only for promoting psychological well-being in older adults but also for designing effective interventions that support identity continuity and life satisfaction. Memory is not a fixed trace; rather, it is a dynamic process that is continually updated in accordance with current emotional states, social contexts, and self-concepts [1,2]. Research in both animals and humans has shown that memories can become temporarily labile upon retrieval and, through reconsolidation, acquire new meanings [3,4]. Autobiographical memory is therefore not merely a repository of past events but a psychological construct that underpins the sense of “who one is” and how the present self has been formed [5]. Consequently, age-related or stress-related changes in memory may affect not only the quantity or accuracy of remembered information but also the sense of self-continuity and the processes through which meaning is constructed.

Recent studies suggest that what is critical for psychological adaptation and self-integration is not simply the amount of memory retained, but how memories are organized into coherent, causally connected narratives [6], although the strength and directionality of this relationship vary across studies. Memories associated with intense emotion may resist integration, leading to fragmented or poorly contextualized recollections [7]. Narratives provide a framework that situates events along temporal axes, assigns causal relationships, and ascribes meaning. Both clinical approaches, such as narrative therapy and reminiscence therapy, and non-clinical activities, including creative practices, expressive writing, and life review exercises, have been shown to influence memory retrieval and emotional transformation through narrative expression [8]. These interventions facilitate autobiographical reasoning, enhance temporal and causal coherence, and contribute to the reconstruction of a stable and adaptive self-concept.

Importantly, narrative processes are inherently social. Memories and self-evaluations are continually updated through interactions with others, particularly when the narrator experiences understanding and empathic engagement. These processes should be interpreted as context-dependent and not uniformly established across all interpersonal settings. This social dimension highlights that self-continuity and psychological adaptation are not solely intrapersonal phenomena but emerge through relational processes in which listeners’ responses shape the content, structure, and meaning of recalled experiences. In the present review, empathy is not treated as a unitary construct. We distinguish between affective empathy, which involves emotional sharing, and cognitive empathy, which involves perspective-taking and mental state inference. The proposed model primarily emphasizes cognitive empathy as a predictive and meaning-making process that supports narrative reconstruction and memory updating. However, this characterization remains theoretical and is not consistently supported across empirical studies, particularly with respect to its predictive function. Empathy, in particular, can function as a psychosocial and emotional regulatory condition that may support the reintegration of fragmented memories, potentially reduce defensive responses, and facilitate reinterpretation of emotionally salient events, although the causal mechanisms underlying these effects remain to be fully clarified.

While narrative interventions have long been employed in psychotherapy, gerontology, and educational contexts to facilitate autobiographical memory integration, identity reconstruction, and psychological adaptation, these approaches have often emphasized the act of retelling or creative expression without consistently or explicitly incorporating the listener’s empathic engagement as an explicit mediating factor. In contrast, the present review focuses on empathy-mediated narrative interventions, in which empathic responses from listeners are deliberately positioned as a core mechanism that supports memory reconsolidation, meaning-making, and self-narrative updating. This distinction is conceptually proposed and requires further empirical validation.

Based on the structured thematic synthesis of the included literature conducted in this review, three recurring conceptual domains were identified across disciplines: (1) the plasticity of autobiographical memory through reconsolidation processes, (2) the role of narrative coherence in meaning-making and self-integration, and (3) the mediating function of empathic social interaction in stabilizing or transforming reconstructed memories, although these domains reflect a conceptual synthesis of heterogeneous evidence rather than uniformly convergent empirical findings.

These domains emerged through iterative comparison and conceptual categorization of findings across psychological, neuroscientific, and narrative research traditions.

Accordingly, the present review integrates these domains and proposes an integrative conceptual model in which empathically grounded narrative practices function as a mechanism for autobiographical memory updating and reconstruction of self-continuity, particularly under conditions of aging or psychological stress.

This model should be understood as a theoretically informed framework rather than a definitive causal account.

## 2. Methods: Literature Selection and Analytical Framework

### 2.1. Review Design and Scope

This study was conducted as a theory-oriented narrative review aimed at organizing and integrating conceptual frameworks related to the reconstruction of autobiographical memory through narrative processes within empathically mediated social contexts.

The objective of the review was theory development rather than quantitative aggregation. Accordingly, the findings should be interpreted as conceptually integrative rather than systematically exhaustive or quantitatively conclusive. Accordingly, the study does not follow the procedural requirements of a systematic review or meta-analysis (e.g., PRISMA flow documentation, exhaustive retrieval, or formal risk-of-bias assessment). Instead, it was designed to ensure conceptual transparency and logical coherence in the literature identification and synthesis.

The primary goal was to synthesize interdisciplinary theoretical perspectives and empirical findings across psychology, neuroscience, psychotherapy, gerontology, and social cognition in order to develop an integrative explanatory model of empathy-mediated narrative reconstruction. Emphasis was therefore placed on conceptual relevance, theoretical contribution, and cross-disciplinary integration rather than comprehensive coverage of all available publications.

### 2.2. Literature Search Strategy

Literature was identified through searches of major international academic databases, including PubMed, PsycINFO, Web of Science, and Scopus. To incorporate relevant Japanese scholarship, J-STAGE and CiNii were additionally consulted.

Initial searches were conducted between March 2024 and October 2025, with additional targeted searches performed iteratively as the conceptual framework evolved. The search primarily focused on studies published from 2000 onward, while earlier foundational works were included when deemed conceptually essential.

Search terms were combined using Boolean operators and included variations in the following keywords: “narrative,” “autobiographical memory,” “memory reconsolidation,” “empathy,” “social interaction,” “retrieval,” “meaning-making,” “expressive writing,” “reminiscence,” and “life review.”

The search process was iterative in that emerging conceptual distinctions informed subsequent targeted searches. The aim was not exhaustive retrieval but the identification of theoretically central and empirically informative studies within each conceptual domain. This approach may introduce selection bias and limit the reproducibility of the search process.

Google Scholar was used as a supplementary tool to trace citation networks and identify additional influential publications. Reference lists of key theoretical papers and relevant reviews were manually examined to ensure that foundational works were incorporated.

To enhance transparency and provide an approximate account of the reproducibility of the literature identification process, the search and selection procedure was retrospectively reconstructed. An initial search across databases yielded approximately 1300 records. After removal of clearly irrelevant titles and duplicates, approximately 190 studies were retained for abstract-level screening. Following further evaluation based on the inclusion and exclusion criteria described below, 54 studies were considered directly relevant to the three focal domains and were included in the final conceptual synthesis.

Although the review was not originally conducted using a predefined systematic protocol, these estimates provide an indicative and transparent account of how the final body of evidence was assembled. The selection process prioritized conceptual relevance and theoretical contribution rather than exhaustive coverage, consistent with the aims of a theory-oriented narrative review.

This approach is consistent with integrative narrative review methodology, in which literature identification proceeds alongside theory refinement.

### 2.3. Inclusion Criteria

Studies were eligible for inclusion if they addressed at least two of the following three conceptual domains as primary analytical components:

1. Narrative construction or narrative practices (e.g., autobiographical narration, life review, expressive writing, storytelling, narrative identity formation).
2. Autobiographical or emotionally salient memory processes (e.g., episodic memory, emotional memory, reconsolidation, memory updating).
3. Empathy or socially mediated processes (e.g., interpersonal interaction, social cognition, empathic engagement, listener–speaker dynamics).

Both theoretical and empirical studies were included, encompassing experimental research, neuroimaging studies, intervention studies, qualitative analyses, and relevant conceptual reviews.

Selection decisions were guided by conceptual centrality rather than study design hierarchy. Studies were included when their theoretical framework or empirical findings directly informed the integrative model developed in this review.

Priority was given to peer-reviewed publications from 2010 onward to reflect recent theoretical and methodological developments. Foundational works published prior to 2010 were retained when they served as essential conceptual anchors for contemporary models of autobiographical memory, narrative identity, or reminiscence.

### 2.4. Exclusion Criteria

Studies were excluded if they met any of the following conditions:

1. Research on memory that did not address narrative generation, narrative organization, or meaning-making processes.
2. Studies employing the concept of “narrative” solely in a metaphorical or descriptive sense without analytical relevance.
3. Case reports lacking broader theoretical integration.
4. Publications without accessible full text.

In addition, purely technical studies focusing exclusively on memory performance without consideration of subjective experience, narrative integration, or social context were excluded.

Exclusion decisions were based on conceptual relevance rather than formal methodological scoring, reflecting the theory-building objective of the review.

### 2.5. Analytical Approach

Selected studies were analyzed using a thematic and conceptual synthesis approach aimed at identifying explanatory linkages across domains.

The analytical procedure involved three sequential steps. First, studies were organized according to their primary contribution to one or more of the three focal domains: narrative processes, autobiographical memory mechanisms, and empathy or social interaction.

Second, theoretical connections and convergent explanatory patterns across domains were examined, with particular attention to recurring mechanisms such as memory destabilization, narrative coherence, empathic modulation, and reconsolidation.

Third, these cross-domain linkages were integrated into a cyclical explanatory framework that forms the basis of the empathy-mediated narrative reconstruction model presented in subsequent sections.

The development of thematic categories was guided by explicit conceptual criteria derived from established theories in autobiographical memory research, narrative identity theory, and social cognition. The aim was deductive–integrative model construction rather than inductive theme generation.

To address potential disagreements and conceptual ambiguities across studies, findings that showed partial inconsistency or divergent interpretations were not excluded but were examined for their contextual and theoretical implications. In cases of conflicting evidence, priority was not assigned solely based on study design but on explanatory relevance and conceptual coherence within the integrative framework.

Borderline cases—such as studies addressing only one or two domains indirectly—were evaluated in terms of their contribution to cross-domain linkage rather than strict categorical inclusion. When conceptual definitions (e.g., empathy, narrative, or reconsolidation) varied across studies, these differences were explicitly acknowledged and interpreted as reflecting heterogeneity in operationalization rather than methodological deficiency.

To minimize confirmatory bias in the synthesis process, the analysis actively considered disconfirming or non-aligned findings and incorporated them as boundary conditions or limitations of the proposed model, rather than excluding them from the framework. This approach was intended to preserve conceptual transparency and prevent overgeneralization.

This analytical strategy prioritizes explanatory coherence and interdisciplinary consistency over statistical aggregation, consistent with the objective of developing a theoretically grounded framework.

To enhance transparency, the empirical and theoretical studies informing the present framework are summarized in Supplementary Table S1.

In the present review, we distinguish between three levels of evidence:

(1) Empirically established findings;
(2) Theoretically plausible linkages;
(3) Integrative extensions proposed in this review.

Future research should aim to validate the proposed framework using systematic and hypothesis-driven designs to strengthen empirical grounding.

## 3. Autobiographical Memory and Narrative Reconstruction

Autobiographical memory has been widely conceptualized as a dynamic and reconstructive system rather than a static repository of past experiences. The reviewed literature suggests that autobiographical memory may function as a plastic, socially embedded system supporting self-continuity, although the strength and consistency of evidence vary across studies and contexts.

At the neurocognitive level, reconsolidation theory provides a potential mechanism for understanding such plasticity. When memories are reactivated, they may re-enter a labile state in which modification becomes possible. However, reconsolidation processes may allow previously stored memories to become modifiable under certain conditions, rather than guaranteeing systematic transformation.

Building on this perspective, the thematic synthesis of the included literature identified autobiographical memory reconstruction as a core domain underlying empathy-mediated narrative processes.

Across psychological, neuroscientific, and narrative research traditions, autobiographical memory was generally conceptualized not as a static record of past events, but as a reconstructive system shaped by retrieval conditions and social context [5], although some studies report variability depending on contextual and methodological factors.

Autobiographical memory is not necessarily retained in the form in which experiences originally occurred; rather, during retrieval, memories are selectively accessed and reorganized in accordance with the individual’s current psychological state and social context [5]. Through this reconstructive process, memories are transformed from fragmented episodes into narratives that integrate temporal order, causal relationships, and emotional meaning. Such narrativization serves the function of assigning meaning to personal experiences and enhancing an individual’s understanding of their life as a whole, as well as their outlook for the future [8]. Findings across disciplines suggest that narrativization may function as a mechanism for integrating temporal order, emotional appraisal, and personal significance, although the strength and nature of evidence vary across research traditions.

Based on this perspective, interventions such as narrative therapy and reminiscence therapy have been implemented to promote psychological stability and the formation of a positive self-image through the retelling of past experiences [9]. More recently, research in psychology and gerontology has proposed integrative approaches that combine narrative and reminiscence, suggesting that reconstructing one’s life as a coherent story and reinterpreting the meaning of past events may enhance a subjective sense of life meaning and self-integration [10]. Rather than viewing these approaches as isolated clinical techniques, the reviewed literature supports interpreting them as applied manifestations of broader reconstructive memory mechanisms identified through thematic synthesis.

### 3.1. Autobiographical Memory and the Self

Within the reviewed literature, autobiographical memory emerged as a central mechanism supporting self-concept integration.

Autobiographical memory is a core psychological function that integrates personal experiences temporally and meaningfully, thereby supporting self-understanding. Theoretical and clinical research has demonstrated that autobiographical memory is not merely a repository of events but plays an active role in forming and reorganizing the self-concept through narrative structure and coherence [5], although the extent and mechanisms of this relationship may vary across individuals and contexts. Accordingly, memory is best understood not as static information that is retrieved intact, but as a dynamic process that is continually updated through retelling.

From a neuroscientific standpoint, retrieved memories are known to become temporarily labile and subsequently undergo reconsolidation. This process requires new protein synthesis and depends on several boundary conditions, including retrieval duration, memory strength, and the time elapsed since encoding [1,2,4,11]. These findings are consistent with the reconstructive account identified in this review, suggesting that autobiographical memory updating may be sensitive to emotional and interpersonal contexts rather than being uniformly determined.

Furthermore, by placing events in temporal sequence and incorporating the narrator’s perspective and evaluations, fragmented sensory traces are transformed into “narrated experiences” [12]. Across the reviewed literature, this transformation was repeatedly described as foundational to the maintenance of self-continuity within socially situated contexts. Because narration presupposes an audience and a social background, autobiographical memory is reorganized not only as a personal experience but also as a socially situated representation.

### 3.2. Emotional Memory and Reactivation

A third line of evidence identified in the review concerns the interaction between emotional arousal and memory reactivation, although this evidence derives from heterogeneous methodological approaches.

Emotionally charged events are more readily recalled, and emotional responses are reactivated each time such memories are retrieved. Research has shown that emotional memories can be transformed depending on the mode of expression and retrieval context, with narrativization providing a critical opportunity for updating emotional meaning [13]. Across the reviewed studies, reflective or creative narration was repeatedly described as facilitating reinterpretation of emotional appraisal.

Neuroscientific studies have demonstrated that negative emotional states selectively impair the integration of associative memory, even when individual sensory elements are strongly retained [7,14]. These findings were interpreted within the review framework as supporting the view that memory-related difficulties may reflect structural integration challenges rather than simple retention deficits. From a reconsolidation perspective, memories that enter a plastic state during retrieval are strongly influenced by emotional states and contextual information present at that time. Negative emotions have been suggested to hinder the reintegration of contextual and relational information, indicating that memory-related difficulties reflect not only memory strength but also the structural form in which memories are reconsolidated [7].

Within this framework, empathic and psychologically safe interpersonal contexts are thought to reduce emotional hyperarousal and facilitate the reintegration of associative memory networks. Accordingly, the empathy-mediated narrative intervention model proposed in this review is presented as a conceptual integration of these converging lines of evidence rather than as an independent empirical claim. Empathy thus functions not only as a psychosocial factor but also as an emotional regulatory condition that may support memory reconsolidation, with converging neuroscientific findings providing indirect support for this interpretation rather than definitive causal evidence.

Finally, narrativization of autobiographical memory is always embedded in social contexts. Narrators implicitly anticipate the presence and reactions of listeners when selecting, sequencing, and interpreting events. Memory reconstruction can therefore be understood as a dynamic process shaped by both intrapersonal cognitive operations and interpersonal conditions.

The following section elaborates on how empathy, as a specific form of social interaction, contributes to narrative generation and memory reintegration.

## 4. Narrative Coherence and Meaning-Making

Narrative has been widely proposed as a mechanism through which individuals organize and interpret autobiographical experiences. The reviewed literature suggests that narrative processes may contribute to the structuring and interpretation of personal memories, although the strength and consistency of empirical support vary across studies and methodological approaches.

At a conceptual level, narrative coherence has been associated with psychological continuity and meaning-making. However, this relationship remains partly theoretical, as empirical findings are heterogeneous and depend on differences in operational definitions, measurement approaches, and study populations.

Within the studies included in this review, narrative coherence was frequently examined in relation to psychological adaptation and self-understanding. Several empirical studies indicate that higher levels of narrative coherence are associated with more adaptive psychological functioning [5,15], although the directionality and underlying mechanisms of this association remain subjects of ongoing investigation.

From a functional perspective, narrative coherence may provide a framework through which individuals organize temporally dispersed experiences, establish causal relationships, and assign emotional meaning. In this sense, narrative can be understood as a structuring process that contributes to the integration of autobiographical memory, rather than as a deterministic mechanism that produces coherence in a uniform manner.

Importantly, narrative construction does not occur solely at the intrapersonal level. A substantial body of research suggests that narratives are co-constructed through interaction with others, particularly in conversational and therapeutic contexts [16]. Within this framework, listener responses—especially those characterized by empathic engagement—may influence how experiences are selected, interpreted, and evaluated during narrative production.

Furthermore, some studies suggest that narratives exhibiting higher coherence are more likely to elicit supportive and affirming social responses, which may in turn contribute to short-term emotional regulation and perceived social support [17]. However, these relationships are not uniformly observed across all contexts and populations, indicating the need for further empirical clarification.

The reviewed literature also includes theoretical models that conceptualize autobiographical memory as a distributed system, in which remembering is shared and reconstructed through interpersonal interaction [18]. From the perspective of the present synthesis, these models converge in suggesting that meaning-making processes are not purely intrapersonal but are embedded within social and relational contexts.

Taken together, these findings support the view that narrative coherence may function as an important—but not exclusively determining—mechanism for organizing autobiographical experience and supporting meaning-making. This interpretation provides a conceptual bridge to the following section, which examines the role of empathy as a specific form of social mediation in narrative reconstruction.

### 4.1. Narration as an Act Directed Toward Others

Within the reviewed studies, narration was consistently conceptualized as an inherently relational act.

Narrative storytelling is fundamentally a social act that presupposes an actual or imagined listener. According to the co-construction model, narrators select events, assign causal relations, and incorporate evaluative perspectives while anticipating listeners’ reactions, thereby adapting narratives to interpersonal contexts [16,19]. Memory is thus not merely reproduced but reorganized in a socially meaningful form.

Within this process, empathy functions not only as emotional support but also as metacognitive feedback, signaling that one’s experience is understandable and legitimate to others. Such feedback facilitates the reevaluation of events and the updating of self-positioning, contributing to the formation of more integrated and coherent self-narratives. Across the reviewed literature, this feedback mechanism was repeatedly described as central to narrative restructuring rather than as a peripheral interpersonal variable.

### 4.2. Empathic Responses and Narrative Structure

A second recurring theme concerned the structural impact of empathic responses on narrative coherence.

Empathic responses involve understanding and accepting another person’s emotions and perspectives. Neuroscientific studies have demonstrated neural alignment between speakers and listeners during storytelling, with listeners’ brain activity becoming temporally synchronized with the speaker’s preceding neural states [20]. This neural coupling suggests that narrative meaning is dynamically aligned and adjusted through interpersonal interaction.

Consistent with these findings, psychological research indicates that empathic, affirming, and attentive responses from listeners directly influence narrative coherence and emotional appraisal [21]. Experimental studies have shown that under empathic listening conditions, narratives become more causally integrated and meaningfully organized. These results suggest that empathy does not simply strengthen memory content quantitatively but instead functions as a regulatory factor that promotes structural reorganization of self-narratives. Across methodological approaches, coherence—not recall quantity—was repeatedly identified as the primary outcome influenced by empathic engagement.

Theoretical perspectives conceptualizing autobiographical memory as a “distributed autobiographical memory system” reconstructed through interaction with others provide an integrative framework for these observations [18]. From this viewpoint, memory is transformed through narration and temporarily stabilized through empathic engagement, highlighting its cyclical and dynamic nature. This convergence of theoretical and empirical findings further supports the classification of empathy as a mediating mechanism within the integrative model proposed in this review.

### 4.3. Social Feedback and Self-Reconstruction

Developmental and educational studies included in the synthesis further demonstrated that narrative identity formation is shaped by structured social feedback.

The narrativization of autobiographical memory develops from early childhood through adolescence and is shaped by parent–child conversations and culturally shared life scripts [22,23]. This developmental trajectory indicates that narrative construction is not only an intrapersonal cognitive process but also a socially and culturally embedded one.

Social feedback to narratives plays a crucial role in updating self-understanding. In educational contexts, evaluative and emotional responses from listeners have been shown to alter narrators’ self-narratives and motivation [24]. These findings suggest that narrative construction operates as a cyclical process involving the individual, others, and broader social contexts, with empathy functioning as a catalyst that drives this dynamic system. Across these domains, empathy consistently emerged as a stabilizing and transformative condition rather than a secondary contextual factor.

### 4.4. Empathy as a Social Condition for Memory Reconstruction

Synthesizing these lines of evidence, empathy can be conceptualized as a core social condition enabling adaptive memory reconstruction.

Taken together, these findings indicate that memory retrieval and narrative construction are inherently social processes shaped by listeners’ presence and empathic responses. Empathy mediates emotional reactivation and meaning-making, serving as a social condition that may support the stabilization or transformation of self-narratives.

Behavioral, electrophysiological, and functional magnetic resonance imaging studies have further demonstrated that autobiographical memory is more strongly engaged during empathy-related tasks than during non-autobiographical memory processing. Evidence of autobiographical memory reactivation within empathy-related neural networks—including the anterior cingulate cortex, parietal regions, and prefrontal cortex—provides strong support for a neural linkage between empathy and memory reconstruction [25].

Accordingly, empathy can be understood not merely as an interpersonal attitude, but as a social and neurobiological condition that guides memory reconsolidation and self-reconstruction. Considered alongside the findings on memory plasticity discussed in Section 3, empathy emerges as a psychosocial context that is firmly grounded in neuroscientific evidence and that facilitates adaptive memory reconstruction.

In summary, narrativization of autobiographical memory is a dynamic process shaped by empathically mediated social interaction. Through understanding and responses from listeners, narrators update their evaluations of events and self-narratives, thereby promoting memory reintegration.

The following section examines how creative narrative activities and narrative interventions, grounded in these social and empathic conditions, influence autobiographical memory reconstruction and psychological well-being.

## 5. Empathy and Social Interaction in Narrative Processes

The thematic synthesis identified empathic social interaction as a third core domain linking autobiographical memory plasticity and narrative coherence. Across the reviewed literature, empathy has been conceptualized not merely as an interpersonal attitude but as a mediating condition that may influence memory reactivation, narrative organization, and self-reconstruction, although the precise causal mechanisms underlying these relationships remain to be fully established.

The narrativization of memory does not occur solely within the individual. Rather, narrators may selectively organize and interpret experiences while anticipating the presence and responses of listeners. Experiences of being understood and empathically engaged during narration have been associated with increased internally focused cognitive processing, as well as changes in self-evaluation and meaning attribution [24]. Through such processes, autobiographical memories may be reinterpreted, contributing to the stabilization or transformation of self-narratives.

From a neuroscientific perspective, this social dimension has been examined in relation to large-scale brain networks such as the default mode network, which has been implicated in autobiographical memory retrieval, future simulation, and perspective-taking [26]. However, these overlapping activation patterns should be interpreted as suggesting—rather than confirming—that narrative processing and empathy may share partially overlapping neural substrates.

Furthermore, some studies suggest that memories that become labile through retrieval and narrativization may be more likely to undergo modification when embedded in supportive interpersonal contexts and accompanied by empathic responses [11]. However, the extent to which such effects reflect causal mechanisms remains an open empirical question.

Taken together, these findings support an interpretation in which empathy may function as a facilitating social condition for narrative reconstruction, rather than as a deterministic mechanism. This perspective provides a conceptual bridge between the reconsolidation processes discussed in Section 3 and the narrative mechanisms outlined in Section 4, contributing to the integrative framework developed in this review.

### 5.1. Empirical Evidence on Autobiographical Memory and Narrative Interventions

A recurring finding across empirical and systematic review studies concerns measurable changes in autobiographical memory structure following narrative-based interventions.

Recent empirical studies and systematic reviews have demonstrated that narrative-based interventions exert significant effects on autobiographical memory retrieval frequency, memory specificity, and meaning reconstruction. A systematic review focusing on adults exposed to trauma or chronic stress reported that narrative interventions reduce memory fragmentation and promote temporally and causally integrated narratives [27]. These effects may not be explained solely by emotional catharsis; rather, they have been interpreted as reflecting changes in autobiographical reasoning processes. This distinction was repeatedly emphasized in the reviewed studies, underscoring that intervention efficacy is linked to structural integration mechanisms.

Autobiographical reasoning plays a central role in maintaining self-identity and psychological continuity through the construction of life stories [28]. Accordingly, interventions that support narrative organization and reinterpretation of past experiences contribute not only to emotional regulation but also to the maintenance and reorganization of self-concept.

Established approaches such as reminiscence therapy and narrative therapy have similarly suggested that retelling past experiences may enhance psychological stability and support the formation of a positive self-image. Importantly, the incorporation of creative elements has been suggested to yield broader psychological and cognitive effects than traditional approaches alone, extending the scope of narrative intervention outcomes [9,10,29]. Across comparative studies, creative augmentation was associated with broader cognitive and affective integration, suggesting additive effects beyond standard reminiscence procedures.

### 5.2. Narrative Quality and Social Responses

The synthesis further revealed that intervention effectiveness depends not only on narrative production but also on narrative quality and interpersonal reception.

The effectiveness of narration depends not only on the content of memories but also on the structural quality of the narrative. Experimental studies have shown that narratives with higher coherence and clarity are more likely to elicit empathic responses and social support from listeners, which in turn is interpreted as reflecting psychological adaptation in narrators [21]. These findings indicate that memory reconstruction is not confined to intrapersonal cognitive processes but is amplified and stabilized through interpersonal interaction.

In addition, the retrieval and retelling of positive autobiographical memories have been shown to promote self-concept and emotional recovery, contributing to the functional reconsolidation of memory through narration. However, counterproductive effects have also been reported when retelling occurs within restrictive belief systems or evaluative frameworks, underscoring the importance of narrative freedom and psychological safety in intervention contexts [30,31]. These findings highlight that social context functions as a moderating condition influencing whether reconstruction leads to adaptive integration or maladaptive reinforcement.

### 5.3. Creative and Expressive Narrative Interventions

Creative and expressive modalities were identified in the reviewed literature as potentially structured mechanisms that may facilitate associative expansion and reinterpretation during memory retrieval.

Growing attention has been directed toward creative narrative activities and storytelling-based interventions aimed at enhancing autobiographical memory, cognitive function, and psychological well-being. Studies involving older adults, individuals with mild cognitive impairment, and those with dementia suggest that interventions incorporating creative elements yield broader benefits than reminiscence therapy alone.

Research using expressive activities such as writing, drawing, and fictional creation has demonstrated greater memory retrieval and emotional integration than simple reproduction of past events [13,32]. Creative reconstruction tasks appear to facilitate the association of retrieved memories with novel meanings, thereby promoting updates to self-narratives. Randomized controlled trials employing Creative Story Therapy have reported significant improvements in cognitive and emotional outcomes, including cognitive function, depressive symptoms, quality of life, and communication abilities, when compared with conventional care [33,34]. Across methodological designs, these outcomes were often interpreted as potentially reflecting enhanced narrative integration and associative flexibility, rather than solely isolated symptom improvement.

### 5.4. An Integrative Model Supported by Empirical Research

Synthesizing these empirical findings, creative narrative interventions can be understood as applied instantiations of the integrative model proposed in this review.

Taken together, these empirical findings support the theoretical framework presented in Section 3 and Section 4, namely, a model of autobiographical memory reconstruction mediated by narrative, empathy, and social feedback. Review studies focusing on digital narrative production have characterized digital storytelling as an intervention that simultaneously promotes memory retrieval, self-expression, and social interaction. In such contexts, the creative act itself functions as a trigger for recall while fostering narrative coherence and social feedback [35].

Furthermore, narrative interventions have been suggested to enhance psychological stability and self-esteem and may contribute not only to cognitive and emotional outcomes but also to broader processes of meaning-making and self-understanding across the lifespan [10,29]. Overall, these findings indicate that the effectiveness of narrative interventions depends on both how stories are constructed and how they are received, and that empathic and psychologically safe social contexts constitute a core condition for enabling memory reconstruction and the updating of self-narratives. This convergence of theoretical models, experimental findings, and intervention studies provides empirical grounding for the integrative framework advanced in the present review.

## 6. Creative Practices and Narrative-Based Interventions

The thematic synthesis further identified creative and narrative-based interventions as applied contexts in which the three core domains—autobiographical memory plasticity, narrative coherence, and empathic social mediation—may converge. Across empirical studies and review articles, these interventions have been examined as structured environments that operationalize narrative reconstruction within socially responsive settings, although reported outcomes and effect sizes vary across populations, methodologies, and intervention designs.

As discussed in the previous sections, the narrativization of autobiographical memory is a dynamic process shaped by socially and empathically mediated interaction. Within such contexts, listener responses may influence how narrators evaluate past experiences and reconstruct self-narratives, potentially facilitating processes of memory reintegration.

Creative narrative activities may act as triggers for the retrieval of autobiographical memory fragments that are not readily accessible under ordinary conditions. These fragments may then be progressively organized through narration and social sharing, acquiring temporal structure and coherence through interpersonal engagement.

Accumulating empirical findings from narrative interventions and reviews of creative and reminiscence-based approaches suggest that such processes may support models of autobiographical memory reconstruction mediated by narrative, empathy, and social feedback. However, these interpretations should be understood as indicative rather than conclusive, given variability in study design and methodological rigor.

Some studies report that these interventions are associated with positive psychological outcomes, including improvements in psychological stability, self-esteem, and perceived meaning in life [27,29]. Nevertheless, these effects are not uniformly observed across all contexts, and intervention outcomes are often interpreted in relation to changes in narrative organization rather than solely in terms of symptom reduction.

Taken together, these findings suggest that creative and narrative-based interventions may provide applied contexts in which the processes described in previous sections can be examined and potentially facilitated, while further empirical validation remains necessary.

**Figure 1:**
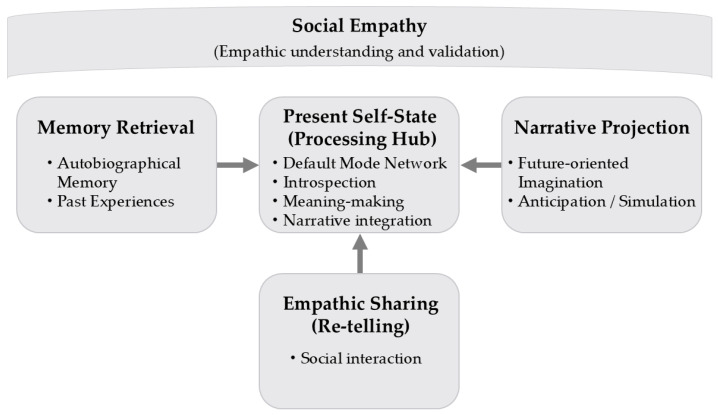
Empathy-mediated narrative reconstruction model.

As shown in Figure 1, the empathy-mediated narrative reconstruction model depicts the cyclical updating of autobiographical memory across past, present, and future dimensions. Autobiographical memory retrieval brings past experiences into the present, where retrieved memories temporarily enter a labile and modifiable state. These memories are processed within the present self-state, conceptualized as a cognitive and neural processing hub involving the default mode network, introspection, meaning-making, and narrative integration. From this present hub, narrative projection supports future-oriented imagination, anticipation, and simulation, enabling the construction of prospective narratives.

Empathic sharing and re-telling represent socially mediated processes through which narratives are selectively expressed, shared, and refined in interaction with others. Social empathy, positioned as an overarching contextual condition, provides psychological safety, mutual understanding, and emotional resonance that facilitate memory reintegration and adaptive narrative updating. The primary cyclical flow links memory retrieval, present self-state processing, narrative projection, and empathic sharing.

The model is presented at a conceptual level, with specific narrative practices and creative activities subsumed within the processes of present self-state processing and empathic social interaction. Together, the model emphasizes how cognitive, social, and narrative processes interact dynamically to support autobiographical memory reconsolidation and the continuous updating of self-narratives.

Systematic reviews of narrative interventions for trauma-related disorders have demonstrated that retelling experiences and reassigning meaning have been associated with reductions in symptoms and the restoration of future-oriented perspectives [27]. These findings suggest that similar mechanisms may support psychological adaptation in older adults and individuals with dementia, populations in which autobiographical memory integration is often compromised [36,37]. The extension of this mechanism across populations may suggest the potential generalizability of the integrative framework.

Creative activities such as expressive writing further support this model. Writing has been reported to be associated with changes in neural activity in reward-related and emotion-related brain regions during subsequent learning, indicating that verbalizing past experiences influences emotional processing beyond the immediate context [29]. Writing-based interventions are also effective in regulating difficult emotions among individuals with interpersonal avoidance or social inhibition, without requiring intensive face-to-face interaction [38]. These findings illustrate that narrative reconstruction mechanisms can operate across varying degrees of interpersonal proximity.

Recent studies employing systematic frameworks to classify narrative elements have clarified how characteristics such as emotional vividness and plot structure influence empathic responses to narratives [39]. Empathy is shaped not only by narrative content but also by modes of narration and expression, and it plays a central role in narrative sharing and meaning-making [40]. Furthermore, discrepancies between anticipated narrative content and the outcomes of narration or creative production may function as prediction errors, thereby increasing memory plasticity and enabling reconsolidation. This prediction-error perspective provides a mechanistic bridge between cognitive neuroscience and narrative intervention research.

### 6.1. Applications in Clinical Contexts: Narrative Interventions and Memory Reintegration

Clinical applications provide structured environments in which the proposed model can be observed and evaluated.

In clinical psychology and psychiatry, narrative therapy and narrative exposure therapy are widely used to facilitate the reintegration of traumatic memories [41,42]. These approaches emphasize temporal and causal reconstruction rather than factual accuracy, allowing fragmented memories to be reorganized and their emotional meanings updated.

Reinterpreting life events from a narrative perspective enables individuals to integrate negative experiences into a continuous sense of identity and to reframe them as part of personal growth [41]. In narrative exposure therapy, retelling experiences within a safe therapeutic context, combined with the therapist’s active engagement as an empathic listener, is thought to strengthen top-down regulation of fear memories [43]. This interaction may illustrate a central mechanism proposed in the model: retrieval within an empathically regulated context enabling adaptive updating.

At the same time, concerns regarding symptom exacerbation due to repeated exposure to traumatic memories highlight the importance of careful clinical judgment. Meta-analyses have demonstrated that narrative exposure therapy effectively repositions traumatic memories within temporal context, reduces intrusive symptoms, and produces sustained long-term effects [42,44]. Neurobiological findings further suggest post-intervention changes in emotional regulation, supporting the notion that the act of “telling” itself may contribute to processes related to memory reconsolidation [43].

In gerontological psychology, reminiscence and life review interventions have been associated with reductions in depressive symptoms and improvements in subjective well-being [36,37]. More recently, creative reminiscence approaches have gained attention for their ability to move beyond simple recall and reconstruct past experiences as new narratives, thereby supporting identity reaffirmation [45]. These findings extend the applicability of the model beyond trauma-focused treatment to aging-related identity processes.

### 6.2. Applications in Educational and Developmental Contexts

Developmental and educational research further supports the model’s relevance beyond clinical settings.

Autobiographical memory is often conceptualized as a hierarchical structure comprising self-defining memories, narrative scripts, and life stories, which together support self-coherence [46]. Through narrative integration, individual experiences are embedded within broader life stories, facilitating self-understanding and identity reconstruction.

In educational settings, reflective writing, autobiographical storytelling, and collaborative narrative activities are commonly used to support emotional regulation and self-reflection [24]. Sharing narratives with others has been shown to deepen meaning-making through empathic understanding and social feedback [21]. These findings suggest that even in non-clinical contexts, the presence of listeners and opportunities for narrative exchange play a crucial role in memory re-signification. Thus, the integrative model captures mechanisms observable across developmental stages and institutional contexts.

### 6.3. Workshop Design Using Creative Activities

Creative workshop formats offer experimentally manipulable environments for examining empathy-mediated reconstruction processes.

Creative workshops incorporating fiction writing, theatrical reenactment, drawing, or collaborative production can indirectly activate autobiographical memory while minimizing the burden of direct self-disclosure. Narratives generated through such activities need not correspond precisely to factual events; emotional memories may be reorganized through imaginative transformation [47].

In these contexts, listener responses function as social scaffolding, enabling narrators to explore alternative meanings and perspectives [47]. Empirical studies integrating art-based participation and reminiscence have shown that creative expression acts as a trigger for memory retrieval and influences both autobiographical memory and emotional experience [48]. These findings illustrate how creative structure and empathic feedback jointly may facilitate memory updating.

**Figure 2:**
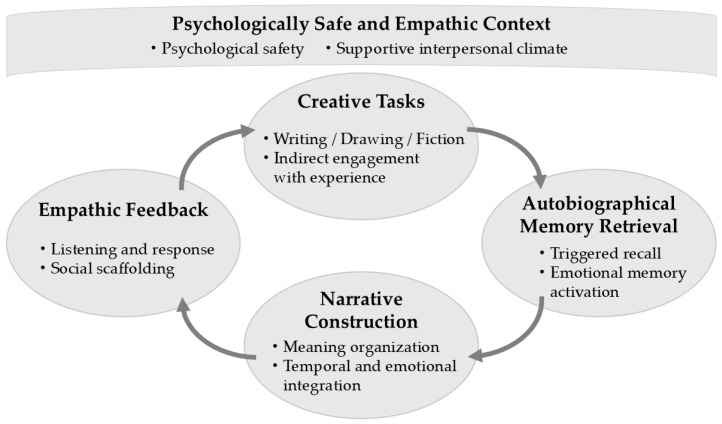
Conceptual model of a creative workshop for empathy-mediated narrative reconstruction.

A conceptual workshop model is illustrated in Figure 2. This model emphasizes (1) the structure of creative tasks, (2) the presence of empathic feedback, and (3) qualitative changes in retrieved memories. Game-based fiction-writing formats, for example, allow feedback roles to shift dynamically, enabling participants to experience social empathy through playful and engaging creative interaction. The model is presented as a conceptual scaffold intended to guide hypothesis-driven empirical testing rather than as a finalized intervention protocol.

As shown in Figure 2, this conceptual workshop model represents a workshop-based implementation of the empathy-mediated narrative reconstruction framework. Creative tasks—such as fiction writing, drawing, theatrical reenactment, and collaborative production—serve as indirect triggers for autobiographical and emotional memory activation while reducing the burden of direct self-disclosure. Retrieved memories enter a malleable state and are transformed through imaginative narrative construction and meaning-making.

Empathic feedback from others, including attentive listening, validation, and perspective-taking, functions as social scaffolding that supports emotional regulation, psychological safety, and narrative refinement. Through this iterative process, qualitative changes in autobiographical memory emerge, including memory reintegration, emotional reappraisal, and the reconstruction of self-narratives. The cyclical structure highlights how creative activity, empathic interaction, and narrative transformation dynamically interact to facilitate adaptive memory updating.

### 6.4. A Proposed Practice Model: Empathy-Mediated Narrative Intervention

Building on the reviewed evidence, a practice-oriented framework grounded in prediction-error theory is proposed.

Based on the reviewed findings, this paper proposes an empathy-mediated narrative intervention model grounded in the principle of prediction error. Memory reconsolidation is thought not to be triggered by recall alone but may occur when discrepancies arise between anticipated memory content and new experiential input [49].

Creative activities generate interpersonal interaction and enable individuals to re-evaluate themselves from third-person perspectives through empathic responses. The creation of new narratives disrupts existing predictions and introduces novel interpretations, producing prediction errors that may facilitate processes associated with memory reconsolidation [50,51].

Within this model, a cyclical process consisting of (1) creation, (2) retrieval, (3) narration, (4) empathic response, (5) retelling or new creation, and (6) memory reconstruction is treated as the basic unit of intervention. Empathy is positioned not merely as an emotional reaction but as a social mechanism that supports narrative structuring and memory stabilization. This formulation operationalizes the integrative framework in terms suitable for experimental and clinical implementation.

### 6.5. From Theory to Practice

The transition from conceptual synthesis to applied design underscores the translational orientation of the present review.

This section has demonstrated how theoretical and empirical insights can be translated into clinical, educational, and creative practices. Acts of telling, writing, and creating stories promote memory reconstruction through narrativization and social sharing, and creative interventions can intentionally activate these processes [52].

At the same time, practical applications require continuous critical evaluation. Nevertheless, the framework outlined here provides a foundation for future hypothesis-driven and mechanism-oriented research [53]. As practices that support the retrieval of forgotten memories and the re-signification of difficult experiences, empathy-mediated narrative interventions warrant further systematic investigation.

In summary, autobiographical memory reconstruction can be understood as a cyclical process extending from recall to psychological adaptation through creative activity, narration, and empathic feedback. The empathy-mediated narrative intervention model proposed here suggests that memory reconsolidation is facilitated through the interaction of creation, retrieval, narration, sharing, and reconstruction. Rather than presenting a definitive theory, the model offers an integrative and testable framework intended to stimulate interdisciplinary empirical research.

The following section situates this model within a broader interdisciplinary context, critically examining empathy as a predictive social function, its consistency with reward learning and neuroscientific findings, and key limitations and future challenges. These applications should be regarded as theoretically informed implications rather than empirically established intervention effects.

## 7. Discussion

The present review integrates findings from psychology, neuroscience, and narrative research to clarify how autobiographical memory is reconstructed through narrativization within empathically mediated social contexts. Importantly, the present review integrates findings with different epistemological statuses, including established empirical evidence, theoretically plausible linkages, and speculative extensions proposed as part of the current framework.

Not all cited findings provide direct evidence for autobiographical memory reconsolidation; some contribute indirect or contextually relevant support and should be interpreted accordingly.

By synthesizing evidence on memory reconsolidation, narrative coherence, and social cognition, this review advances a theoretical framework in which memory updating and self-narrative reconstruction are understood as cyclical and socially embedded processes. Rather than conceptualizing memory as a purely intrapsychic phenomenon, this framework emphasizes its relational and interactive dimensions. In the following sections, we first interpret the proposed empathy-mediated narrative reconstruction model in relation to existing theories of memory and identity (Section 7.1), then reconceptualize empathy as a future-oriented predictive function (Section 7.2 and Section 7.3), and finally discuss reciprocity, neurobiological consistency, limitations, and directions for future research (Section 7.4, Section 7.5, Section 7.6 and Section 7.7).

### 7.1. An Integrative Model: Empathy-Mediated Narrative Reconstruction

The integrative model presented in this section synthesizes the three core domains identified through thematic analysis: autobiographical memory plasticity, narrative coherence, and empathic social mediation. Rather than introducing a novel speculative construct, the model integrates converging theoretical and empirical findings reviewed in the preceding sections, while acknowledging variability in the strength and consistency of available evidence.

Narrative-based memory interventions may be particularly relevant for populations in whom the retrieval and integration of autobiographical memory have become fragile due to aging, illness, or psychological stress. Narrative practices may provide a framework for psychological adaptation and self-understanding by supporting the reinterpretation of past experiences and their integration into coherent self-narratives [27,28], although the extent of these effects varies across contexts.

Importantly, autobiographical memory reconstruction does not occur solely through intrapersonal cognitive processes. Rather, it may unfold as a dynamic process shaped by interaction with listeners and empathic responses. Across the reviewed literature, such social mediation has been described as an enabling condition for memory reintegration, although the strength and interpretation of this evidence vary across studies and contexts.

Against this background, the present review proposes an empathy-mediated narrative reconstruction model (Figure 1). The model conceptualizes autobiographical memory reconstruction as a cyclical process involving: memory retrieval → narrative construction → partial retelling → empathic social interaction → self-reconstruction → the emergence of revised narratives and identity. This formulation reflects patterns identified across narrative research, intervention studies, and neuroscientific findings. However, this convergence should be interpreted cautiously, as it reflects conceptual alignment across heterogeneous methodologies rather than uniform empirical validation.

Some studies suggest that highly distressing memories may be avoided or defensively suppressed, limiting engagement in conventional narrative recall. In contrast, supportive and empathically mediated contexts may facilitate engagement and reinterpretation processes. Reviews of narrative-based interventions for trauma-related conditions report associations between narrative reconstruction and reductions in psychological distress, as well as improvements in future-oriented perspectives [27]. However, these findings do not establish uniform effects across all populations.

These mechanisms may also be relevant for older adults and individuals with cognitive decline, in whom autobiographical memory integration is often compromised [36,37]. However, the generalizability of these effects across populations remains an open empirical question.

Creative practices such as expressive writing have also been examined as potential facilitators of narrative reconstruction. Some studies report associations between expressive writing and changes in neural activity in emotion- and reward-related regions during subsequent processing [29], as well as improvements in emotional regulation among individuals with interpersonal avoidance [38]. These findings suggest that narrative reconstruction processes may operate across varying levels of interpersonal engagement.

Recent studies examining narrative structure and emotional expression indicate that features such as emotional intensity and plot organization may influence empathic responses to narratives [39,40]. In addition, discrepancies between expected and actual narrative outcomes have been discussed in relation to prediction error mechanisms, which may contribute to memory updating and reconsolidation processes. This perspective provides a potential conceptual bridge between cognitive neuroscience and narrative intervention research.

Taken together, the proposed model should be understood as a preliminary integrative framework that organizes existing findings and generates testable hypotheses, rather than as a fully validated causal account.

### 7.2. Integrative Interpretation: A Cyclical Model of Narrative, Empathy, and Memory Reconstruction

This review examined how acts of telling, writing, and creative narrative production facilitate the retrieval and reconstruction of autobiographical memory and contribute to the updating of self-narratives, drawing on theoretical, empirical, and applied perspectives. Autobiographical memory is not a static record of the past but a dynamic system that is reconstructed each time it is recalled or narrated, with narrative meaning-making serving as its central organizing principle. In this sense, narrativization functions as both a cognitive structuring process and a socially mediated meaning-making activity.

A key conclusion of this review is that memory reconstruction is not confined to intrapersonal cognitive processes. Rather, it may be facilitated and stabilized through social interaction, particularly through mutual understanding and empathic responses from listeners. Narrative structure and coherence elicit comprehension and empathy, and this social feedback, in turn, reshapes the narrator’s interpretation of past experiences and self-concept. Through this iterative exchange, autobiographical memory becomes progressively reorganized in ways that reflect both personal reflection and interpersonal validation. This reciprocal and cyclical process is proposed as a central mechanism within the present integrative framework, although this interpretation should be understood as a theoretically informed synthesis rather than a uniformly established empirical conclusion, given variability in the strength and consistency of available evidence.

### 7.3. Redefining Empathy: Social Cognition as Future-Oriented Prediction

Within this framework, empathy is reconceptualized not merely as emotional resonance but as a cognitive and predictive process. In the present framework, empathy is conceptualized as comprising at least two partially dissociable components: affective empathy (emotional sharing) and cognitive empathy (perspective-taking and predictive simulation). The current model primarily emphasizes the latter. Empathy involves imagining another person’s perspective and situation and internally simulating how one would feel and act under similar conditions. From this viewpoint, anticipating others’ responses and one’s own future reactions is a fundamental component of empathic understanding. In this sense, empathy entails prospective simulation grounded in autobiographical memory and prior social learning.

Empathy can therefore be understood as an advanced form of social cognition that may draw upon past experiences and autobiographical memory to guide future-oriented behavior. Rather than being limited to the sharing of present emotions, empathy functions as a predictive mechanism that informs relationship formation and behavioral decision-making, thereby linking memory research with broader models of social cognition. This predictive dimension positions empathy at the intersection of memory, imagination, and social adaptation.

However, this predictive account should be considered a theoretical proposition rather than a uniformly established empirical finding, as supporting evidence remains heterogeneous across studies.

### 7.4. Empathy and Reward Learning: Implications for Motivation and Relationship Formation

This review further proposes a theoretical hypothesis regarding the motivational basis of empathic processes from a developmental and learning perspective. Experiences of being empathically understood activate neural reward systems, suggesting that “being understood” may be experienced as a rewarding outcome and may motivate individuals to seek further empathic relationships [54]. Such reinforcement processes may contribute to the stabilization of empathically attuned social networks across development.

Within this framework, eliciting empathy from others requires narratives to be structured in a manner that is comprehensible and meaningful to listeners—namely, as temporally and causally coherent stories. When a narrative is understood, the narrator experiences positive affect associated with validation, while the listener experiences satisfaction linked to successful understanding and empathic engagement. This bidirectional reinforcement strengthens interpersonal bonds, supports trust formation, and guides the selection of cooperative behaviors. Accordingly, empathy is proposed to function as both an interpretive mechanism for past experience and a motivational system shaping future social interaction. These interpretations should be understood as theoretical extensions rather than empirically established causal mechanisms.

### 7.5. The Reciprocity of Narrative Communication

Narrative communication not only promotes memory reconstruction in narrators but may also influence the listener’s own memory processes and meaning-making. Receiving a narrative empathically can activate listeners’ autobiographical memories, triggering new associations and personal reinterpretations. Thus, empathic listening may also contribute to processes consistent with memory reconsolidation in the listener, although direct evidence remains limited. Therefore, these processes should be interpreted cautiously pending further empirical validation.

From this perspective, narrative communication is not a unidirectional transmission of experience but a reciprocal process in which memory reconstruction may occur in both participants. Narratives function simultaneously as internal representations of lived experience and as social media through which meaning is co-constructed via interpersonal interaction. This reciprocity underscores the fundamentally intersubjective character of autobiographical memory reconstruction.

### 7.6. Neurobiological Consistency: Links to Emotion and Memory Reconsolidation

The proposed model is consistent with neuroscientific findings on emotion and memory reconsolidation. Emotional states are known to influence reconsolidation processes, and intense negative emotions, in particular, can disrupt associative and contextual integration of memories [7]. These findings suggest that emotional regulation is not peripheral but central to adaptive memory updating.

These findings suggest the potential importance of empathic and psychologically safe interpersonal contexts as conditions for effective narrative reintegration of fragmented memories. Empathy therefore operates not only as a psychosocial factor but also as a neurobiological condition that may support memory reconstruction by modulating emotional and cognitive processing. By attenuating excessive arousal and facilitating reflective processing, empathic contexts may increase the likelihood of adaptive reconsolidation. However, these interpretations are based on indirect evidence and should be considered provisional rather than definitive conclusions.

### 7.7. Critical Considerations: Counterarguments and Cautions Regarding Empathy Models

Despite its explanatory value, the present model warrants careful scrutiny [53]. Excessive emotional resonance may lead to empathic distress or burnout, particularly in caregiving and educational settings, and does not necessarily promote growth or adaptive change. Empathy, therefore, cannot be assumed to yield uniformly beneficial outcomes across contexts.

Moreover, empathy comprises multiple components, including cognitive and affective empathy, which may involve distinct neural mechanisms and social functions. The conception of empathy as future-oriented prediction proposed in this review aligns more closely with cognitive empathy and should not be conflated with affective empathy. Failure to distinguish these components risks overgeneralizing the effects of empathy in narrative contexts. Conceptual precision is thus essential for both theoretical refinement and empirical testing.

In addition, narrative coherence and empathic engagement are not inherently ethical or adaptive. While coherent narratives can strongly engage listeners, they may also reduce critical evaluation. The relationship between empathy and narrative construction is therefore not value-neutral and must be examined within broader ethical and social frameworks. Future scholarship should address how power dynamics, cultural norms, and institutional contexts shape the operation of empathy in narrative settings.

Future research should systematically examine these factors under controlled conditions to clarify boundary conditions and improve the empirical grounding of the proposed framework.

### 7.8. Limitations and Future Directions

Several limitations should be acknowledged. The present model should be understood as a heuristic and integrative framework rather than a definitive causal account. First, this review does not directly verify memory reconsolidation through neurophysiological or pharmacological measures within creative or narrative interventions. Reconsolidation is employed here primarily as a theoretical framework rather than as a directly verified neurobiological process within the reviewed interventions. Accordingly, empirical validation of the proposed mechanisms remains an important priority.

Second, the narrative review approach integrates findings across diverse populations and methodologies, limiting direct comparison of effect sizes and causal inference. Constructs such as empathy and social feedback are operationalized heterogeneously across studies, posing challenges for unified quantification. Standardized operational definitions and measurement frameworks will be necessary to advance cumulative research.

Future research should aim to systematically examine the mechanisms proposed in the present framework through rigorous and well-controlled experimental designs. In particular, studies are needed that manipulate key components of the model, including narrative structure, the characteristics of creative tasks, and the presence or absence of empathic feedback during narrative sharing processes.

To advance beyond conceptual integration, it is essential to incorporate mechanism-oriented approaches using neurophysiological and behavioral measures. Techniques such as functional magnetic resonance imaging, functional near-infrared spectroscopy, and electroencephalography may help clarify the neural dynamics underlying empathy-mediated narrative reconstruction and memory updating.

In addition, longitudinal designs are required to examine how narrative processes and empathic interactions contribute to identity development and psychological adaptation over time. Cross-cultural investigations will also be important for evaluating the generalizability and boundary conditions of the proposed framework across diverse social and cultural contexts.

Importantly, future studies should distinguish between direct and indirect evidence related to autobiographical memory reconsolidation and explicitly test the hypothesized mechanisms proposed in this review. Such efforts will be critical for transforming the present heuristic and integrative model into a more empirically grounded and mechanistically specified framework.

## 8. Conclusions

This review aimed to integrate theoretical, empirical, and applied perspectives on autobiographical memory, narrative processes, and empathy into a unified conceptual framework. By proposing the empathy-mediated narrative reconstruction model, the present work highlights how autobiographical memory is dynamically shaped through narrative construction and socially embedded interactions.

Rather than representing a definitive causal account, the proposed model should be understood as a heuristic and integrative framework that organizes diverse findings across disciplines. The synthesis presented here underscores the importance of considering memory as a reconstructive, relational, and future-oriented process, shaped by both internal cognitive mechanisms and external social contexts.

Overall, this framework provides a conceptual foundation for interdisciplinary research and offers a basis for further theoretical refinement and empirical investigation.

## Data Availability

No new data were created or analyzed in this study.

## Supplementary Materials

The following supporting information can be downloaded at: https://www.mdpi.com/article/10.3390/brainsci16040429/s1.
