# Supplementary material for: Empathy-Mediated Narrative Reconstruction of Autobiographical Memory: An Integrative Review of Theory, Evidence, and Applications
Source: Brain Sci. 2026 Apr 20;16(4):429. doi: 10.3390/brainsci16040429 (PMC13114738; doi:10.3390/brainsci16040429)
Supplement: Supplementary file 1 [file brainsci-16-00429-s001.zip › brainsci-4164515-supplementary.pdf]

Supplementary Table S1

Study	Study Type	Key Findings	Relevance to the Present Model
Nader et al., 2000 [1]	Primary empirical study – Experimental (animal)	Retrieved memories require protein synthesis for reconsolidation.	Foundational evidence that reactivated memories become labile and modifiable.
Wang et al., 2005 [2]	Primary empirical study – Experimental (animal)	The amygdala is required for reward memory reconsolidation.	Demonstrates that emotional memories can be updated after reactivation.
Hupbach et al., 2007 [3]	Primary empirical study – Experimental (human – behavioral)	Reminders trigger episodic memory updating.	Supports reminder-based narrative updating.
Kida, 2020 [4]	Secondary research – Narrative review	Identifies boundary conditions for reconsolidation.	Clarifies when memory recall induces plasticity.
Singer and Bonalume, 2010 [5]	Primary empirical study – Clinical case study	Narrative change is associated with symptom improvement.	Suggests that narrative restructuring relates to self-change.
Vanderveren et al., 2017 [6]	Primary empirical study – Cross-sectional study	Narrative coherence is positively associated with well-being.	Supports coherence as adaptive integration.
Bisby and Burgess, 2014 [7]	Primary empirical study – Experimental (human – behavioral)	Emotion enhances central details but weakens contextual details.	Suggests that emotion influences memory structure.
Dings and Newen, 2023 [8]	Conceptual work – Theoretical / Conceptual paper	The narrative self organizes episodic memory.	Memory is structured by self-concept.
Butler, 1963 [9]	Conceptual work – Theoretical / Conceptual paper	Life review facilitates ego integration.	Early theoretical foundation for reminiscence integration.
Bohlmeijer et al., 2008 [10]	Primary empirical study – Clinical intervention study	Reminiscence interventions improve meaning in life.	Narrative re-authoring enhances meaning construction.
Kim et al., 2021 [11]	Primary empirical study	Reconsolidation requires specific boundary	Clarifies conditions necessary for memory

	– Experimental (human – behavioral)	conditions; prediction error is necessary for updating.	reactivation and modification.
Conway and Pleydell-Pearce, 2000 [12]	Conceptual work – Theoretical / Conceptual paper	Autobiographical memory is constructed within a self-memory system.	Frames autobiographical memory as a structured narrative system.
Waters and Fivush, 2014 [15]	Conceptual work – Theoretical / Conceptual paper	Narrative coherence predicts identity integration and psychological adjustment.	Theoretically supports coherence in storytelling.
Hall and Powell, 2011 [16]	Conceptual work – Theoretical / Conceptual paper	A receptive listener facilitates safe narrative expression and emotional activation.	Empathic listening enhances safety in narrating difficult memories.
Vanaken et al., 2021 [17]	Conceptual work – Theoretical / Conceptual paper	Coping with adversity promotes future adaptive coping.	Narrative restructuring contributes to resilience.
Fabry, 2023 [18]	Primary empirical study – Experimental (human – behavioral)	Autobiographical memory is reconstructed through social interaction.	Memory is transformed through narration and stabilized via empathy.
Talarico, 2022 [13]	Secondary research – Narrative review	Imagery and narrative expression enhance memory access.	Creative expression facilitates retrieval of inaccessible memories.
Svoboda et al., 2006 [14]	Secondary research – Narrative review	Autobiographical memory is supported by large-scale networks centered on the default mode network.	Provides neural basis for autobiographical recall.
Shalom and Gross, 2022 [24]	Conceptual work – Theoretical / Conceptual paper	Narrative presentation enhances empathy and memory retention.	Addresses the sequence: narrative → empathy → memory.
Buckner et al., 2008 [26]	Secondary research – Narrative review	The default mode network supports autobiographical and self-referential thought.	Neural foundation of narrative memory.
Lucchi Basili and Sacco,	Conceptual work –	Fiction indirectly promotes behavioral change.	Creative storytelling allows indirect self-disclosure.

2021 [19]	Theoretical / Conceptual paper		
Chang et al., 2024 [20]	Primary empirical study – Experimental (human – neuroimaging)	Neural synchrony predicts interpersonal understanding.	Empathic alignment supports narrative sharing.
Vanaken et al., 2020 [21]	Primary empirical study – Experimental (human – neuroimaging)	Narrative coherence facilitates empathy and understanding.	Coherent narratives enhance social sharing and evaluation.
Wang, 2011 [22]	Conceptual work – Theoretical / Conceptual paper	Autobiographical memory develops within cultural contexts.	Emphasizes culturally contextualized narrative models.
Fivush, 2011 [23]	Conceptual work – Theoretical / Conceptual paper	Autobiographical memory develops coherence through social interaction.	Narrative integration supports self-formation.
Meconi et al., 2021 [25]	Primary empirical study – Experimental (human – neuroimaging)	Autobiographical recall activates empathy-related circuits.	Demonstrates memory–empathy neural interaction.
Raeder et al., 2023 [27]	Secondary research – Meta-analysis	Narrative exposure therapy is effective for post-traumatic stress disorder.	Clinical relevance of narrative updating.
DiMenichi et al., 2019 [29]	Primary empirical study – Experimental (human – neuroimaging)	Emotional expression alters neural processing during learning.	Verbalizing past events modifies neural activity.
Habermas and Camia, 2015 [28]	Conceptual work – Theoretical / Conceptual paper	Autobiographical reasoning maintains self-continuity.	Empathic dialogue mediates autobiographical reasoning.
Moscovitch et al., 2023 [30]	Secondary research – Narrative review	Positive autobiographical recall influences cognition and emotion.	Narrative reconstruction can intentionally shape memory.
Ikeda and Nihei, 2009	Secondary research –	Memory is reconstructive and influenced by	Reframing past experiences transforms memory

[31]	Narrative review	retelling.	representation.
Sugimori et al., 2024 [32]	Primary empirical study – Clinical intervention study	Compassionate letter writing alters memory recall.	Narrative writing fosters empathy toward the past self.
Ma et al., 2023 [33]	Primary empirical study – Clinical intervention study	Creative story therapy improves social communication outcomes.	Supports added value of narrative-based interventions.
Phillips et al., 2010 [34]	Primary empirical study – Longitudinal study	Creative interventions improve emotion and quality of life over time.	Artistic expression indirectly activates empathic processes.
Zhu et al., 2024 [35]	Primary empirical study – Clinical intervention study	Improvements in memory and verbal expression enhance communication.	Empathic sharing supports social reintegration.
Westerhof and Bohlmeijer, 2014 [36]	Secondary research – Narrative review	Reminiscence and life review support mental health.	Integrates autobiographical and narrative research.
Zhong et al., 2023 [37]	Secondary research – Meta-analysis	Effects of reminiscence therapy depend on intervention design.	Structured reminiscence enhances therapeutic benefit.
Ruini and Mortara, 2021 [38]	Secondary research – Narrative review	Reviews expressive writing and narrative therapy techniques.	Narrative interventions promote self-understanding.
Shen et al., 2024 [39]	Primary empirical study – Experimental (human – neuroimaging)	Narrative style influences empathy elicitation.	Empathy arises through stylistic features.
Bal and Veltkamp, 2013 [40]	Primary empirical study – Experimental (human – behavioral)	Narrative immersion increases empathy.	Supports narrative as empathy training.
Lilgendahl and McAdams, 2011 [41]	Primary empirical study – Longitudinal study	Constructing growth narratives predicts well-being over time.	Narrative reconstruction mediates well-being.
Robjant and Fazel, 2010 [42]	Secondary research – Narrative review	Narrative exposure therapy reduces symptoms of post-traumatic stress disorder.	Organizes traumatic memories chronologically.

Adenauer et al., 2011 [43]	Primary empirical study – Experimental (human – neuroimaging)	Narrative exposure therapy is associated with top-down cortical regulation.	Neural mechanisms shared with narrative-based methods.
Siehl et al., 2020 [44]	Primary empirical study – Clinical intervention study	Narrative exposure therapy shows sustained clinical benefits.	Empirical support for narrative-based approaches.
Kenyon and Randall, 1999 [45]	Conceptual work – Theoretical / Conceptual paper	Narrative metaphor informs aging research and practice.	Narrative perspective supports meaning-making in aging.
McAdams and McLean, 2013 [46]	Conceptual work – Theoretical / Conceptual paper	Autobiographical memory underlies self-understanding.	Empathic acceptance supports life-story reconstruction.
Hass-Cohen and Clay, 2025 [47]	Conceptual work – Theoretical / Conceptual paper	Proposes art therapy as inducing reconsolidation processes.	Art therapy enables safe emotional re-experiencing.
Healy et al., 2025 [48]	Primary empirical study – Clinical intervention study	Art combined with reminiscence benefits dementia care.	Art-based narration fosters empathic environments.
Sevenster et al., 2013 [49]	Primary empirical study – Experimental (human – behavioral)	Prediction error is necessary for reconsolidation.	The necessity of prediction error in humans was also theoretically inferred.
Sinclair et al., 2021 [50]	Primary empirical study – Experimental (human – neuroimaging)	Hippocampal prediction error signals drive updating.	Neural mechanism of mismatch detection.
Pupillo et al., 2023 [51]	Primary empirical study – Experimental (human – behavioral)	Prediction error promotes hippocampal updating.	Supports safe prediction-error generation mechanisms.
de Muijnck, 2022 [52]	Conceptual work –	Trauma narratives can be reorganized within	Empathy supports identity reconstruction.

	Theoretical / Conceptual paper	narrative frameworks.	
Strang, 2024 [53]	Secondary research – Narrative review	Critically evaluates art therapy–neuroscience integration.	Provides cautious theoretical positioning.
Kawamichi et al., 2013 [54]	Primary empirical study – Experimental (human – neuroimaging)	Empathic joy activates reward-related neural circuits.	Empathy-based helping suggests a potential mediating role in motivational processes.
